# Advances in Audio Classification and Artificial Intelligence for Respiratory Health and Welfare Monitoring in Swine

**DOI:** 10.3390/biology15020177

**Published:** 2026-01-18

**Authors:** Md Sharifuzzaman, Hong-Seok Mun, Eddiemar B. Lagua, Md Kamrul Hasan, Jin-Gu Kang, Young-Hwa Kim, Ahsan Mehtab, Hae-Rang Park, Chul-Ju Yang

**Affiliations:** 1 Animal Nutrition and Feed Science Laboratory, Department of Animal Science and Technology, Sunchon National University, Suncheon-si 57922, Republic of Korea; baushossain@gmail.com (M.S.); mhs88828@nate.com (H.-S.M.); eddiemarlagua@gmail.com (E.B.L.); kamrul.ps@sau.ac.bd (M.K.H.); kookoo5018@naver.com (J.-G.K.); ahsanmehtab50@gmail.com (A.M.); gofkd20@naver.com (H.-R.P.); 2 Department of Animal Science and Veterinary Medicine, Gopalganj Science and Technology University, Gopalganj 8105, Bangladesh; 3 Department of Multimedia Engineering, Sunchon National University, Suncheon-si 57922, Republic of Korea; 4 Interdisciplinary Program in IT-Bio Convergence System (BK21 Plus), Sunchon National University, Suncheon-si 57922, Republic of Korea; 5 Department of Poultry Science, Sylhet Agricultural University, Sylhet 3100, Bangladesh; 6 Interdisciplinary Program in IT-Bio Convergence System (BK21 Plus), Chonnam National University, Gwangju-si 61186, Republic of Korea; yhkim8760@naver.com; 7 School Education Department, Narowal 51600, Pakistan

**Keywords:** pig vocalization, respiratory disease, cough detection, audio classification, deep learning, edge AI, precision livestock farming, swine health monitoring

## Abstract

Respiratory diseases remain one of the most significant health challenges in modern swine production, leading to substantial economic losses, compromised animal welfare, and increased antimicrobial use. In recent years, advances in artificial intelligence (AI), particularly machine learning and deep learning, have enabled the development of non-invasive, continuous monitoring systems based on pig vocalizations. Among these, audio-based technologies have emerged as especially promising tools for early detection and monitoring of respiratory disorders under real farm conditions. This review provides a comprehensive synthesis of AI-driven audio classification approaches applied to pig farming, with focus on respiratory health and welfare monitoring. First, the biological and acoustic foundations of pig vocalizations and their relevance to health and welfare assessment are outlined. The review then systematically examines sound acquisition technologies, feature engineering strategies, machine learning and deep learning models, and evaluation methodologies reported in the literature. Commercially available systems and recent advances in real-time, edge, and on-farm deployment are also discussed. Finally, key challenges related to data scarcity, generalization, environmental noise, and practical deployment are identified, and emerging opportunities for future research including multimodal sensing, standardized datasets, and explainable AI are highlighted. This review aims to provide researchers, engineers, and industry stakeholders with a consolidated reference to guide the development and adoption of robust AI-based acoustic monitoring systems for respiratory health management in swine.

## Simple Summary

Respiratory diseases are a major challenge in pig farming and can cause serious economic losses if not detected early. Traditional diagnosis relies on visual inspection and laboratory testing, which are time-consuming and often identify problems only after disease has spread. In recent years, artificial intelligence (AI)-based sound analysis has emerged as a promising, non-invasive approach for monitoring pig health by automatically detecting changes in vocalizations such as coughing. This review summarizes recent advances in audio-based monitoring systems for respiratory disease detection and behavior monitoring in pigs, covering microphone technologies, signal processing techniques, machine learning and deep learning models, and on-farm deployment using edge and embedded devices. The strengths, limitations, and practical challenges of current systems are discussed, along with future opportunities for improving accuracy, robustness, and real-time application. AI-driven acoustic monitoring has strong potential to support early disease detection, improve animal welfare, and enhance decision making in modern pig production.

## 1. Introduction

Automatic monitoring systems in pig farming enable continuous, non-invasive observation of individual and group behaviors, providing real-time data that supports timely interventions, reduces labor demands, optimizes environmental conditions, and enhances productivity [1,2,3,4]. These systems use technologies such as cameras, microphones, accelerometers, and sensors to track activities like feeding, drinking, standing, vocalization, and movement, allowing early detection of health issues, stress, or behavioral changes that may indicate disease or poor welfare [5,6]. With advanced machine learning and deep learning algorithms improving detection and behavior classification, these systems facilitate precise health and welfare assessments while helping balance economic goals with sustainable and animal-friendly production [7,8,9].

Acoustic monitoring in pig farming is non-invasive, collects continuous data without disturbing animals, offering clear advantages over vision and wearable sensors particularly for tracking respiratory conditions and detecting environmental stressors such as poor air quality or heat [10]. Unlike vision systems that depend on lighting and clear line-of-sight, acoustic sensors function reliably across diverse environments and can detect subtle vocalizations related to health and welfare; moreover, unlike wearable sensors, they require no physical attachment, avoiding animal stress and reducing labor for maintenance [1,8]. Although challenges remain in achieving high accuracy across different breeds and farm setups, AI and machine learning are improving sound-based interpretation for more reliable health and behavior monitoring [11].

Several review articles have addressed the broader application of artificial intelligence in smart agriculture and precision livestock farming. However, despite the growing number of studies on sound-based monitoring in pigs, a focused and comprehensive review targeting AI-driven respiratory health and welfare monitoring in swine remains lacking. Given the substantial economic and welfare impacts of respiratory disorders in pig production, such a targeted synthesis is timely and necessary.

Accordingly, this review first outlines the biological and emotional basis of pig vocalizations relevant to respiratory tract and social interactions. It then systematically reviews recent studies published on machine learning and deep learning techniques to pig audio data for respiratory health and welfare monitoring, critically comparing model architectures, feature engineering strategies, and reported performance. Commercially available systems and real-time, on-farm deployment solutions are also examined. Finally, this review discusses key limitations of current AI-based audio monitoring technologies and proposes recommendations and future research directions to improve robustness, generalizability, and practical adoption in commercial pig farming.

## 2. Source of Pig Sounds in Farm Environments

### 2.1. Physiological Mechanisms

Pig sounds in farm environments originate from well-defined physiological mechanisms involving the vocal production system and its neural control. Vocalizations are produced when airflow from the lungs passes through the larynx, causing the vocal folds—and in some cases the ventricular folds—to vibrate, while the vocal tract, including the mouth and nasal passages, filters the sound and shapes its acoustic characteristics [12,13,14]. According to the source–filter theory, the vibrating vocal folds act as the sound source that determines the fundamental frequency, whereas the configuration of the vocal tract functions as a filter that modifies resonance and formant structure [13,14,15]. Emotional and physiological states such as arousal, muscle tension, and respiratory patterns influence both the source and filter components, resulting in changes in pitch, duration, and harmonic structure of the calls. Neural and autonomic regulation plays a key role in this process, as emotional arousal alters respiration, heart rate, and muscular activity, which in turn directly affect vocal output; higher arousal levels are typically associated with increased call intensity and higher frequencies, particularly in distress-related vocalizations [15,16,17].

### 2.2. Behavioral and Emotional Contexts

Beyond physiological processes, pig vocalizations are strongly shaped by behavioral and emotional contexts, with distinct call types serving specific functions. Pigs produce a diverse repertoire of calls, including grunts, squeals, and screams, each linked to particular situations and affective states. Grunts are most commonly emitted during neutral or positive contexts such as social interaction and exploration and are generally shorter in duration and lower in pitch when associated with positive emotional states [13,18,19,20,21]. In contrast, squeals and screams are high-frequency, longer-duration calls that typically occur in negative or high-arousal situations, including distress, restraint, or pain [15,21,22]. Both the call type and its acoustic structure—such as frequency, duration, and noisiness—encode information about emotional valence and arousal, with positive emotions producing shorter, lower-pitched sounds and negative emotions generating longer, higher-pitched, and noisier calls [18,20,23]. Additionally, pig vocalizations can reflect social and anticipatory contexts, as spectral and temporal features vary depending on expected outcomes, such as anticipation of rewards or social reunions with conspecifics or humans [18,23].

## 3. Types of Pig Vocalizations

Pig farms are acoustically complex environments characterized by a wide range of sounds that reflect animal health, behavior, welfare status, and routine farm operations. The major categories of sounds include respiratory sounds such as coughs and sneezes, behavioral and activity-related vocalizations including grunts, squeals, screams, and barks, and various environmental noises originating from equipment, ventilation systems, and human activities. These acoustic signals serve as valuable indicators for welfare monitoring, disease detection, and management decision making in pig production systems [24,25,26]. With recent advances in sound-based monitoring technologies and machine learning approaches, it has become increasingly feasible to automatically classify and interpret these sounds, enabling early detection of health problems and more objective assessment of pig welfare [27,28,29,30]. A clear understanding of the types and acoustic characteristics of sounds present in pig farms is therefore essential for both research applications and practical implementation of precision livestock farming tools.

Among farm sounds, respiratory and behavioral vocalizations are particularly informative for health and welfare assessment. Respiratory sounds mainly include coughing, sneezing, and abnormal breathing patterns, with coughing being the most prominent and widely used indicator of respiratory disease in pigs [31,32,33]. Sneezing and labored breathing are also observed, especially during disease outbreaks or under poor air quality conditions and may contribute to early detection of infections such as influenza [11,34,35,36]. In parallel, pigs produce a rich repertoire of behavioral and activity-related vocalizations that reflect their emotional and physiological states. Low-frequency grunts are commonly associated with neutral or positive contexts such as feeding, exploration, and social contact [20,37], whereas high-frequency squeals and screams are typically linked to negative states, including pain, distress, or aggression during handling or fighting [38,39]. Less frequent barks and howls may occur during alarm or intense arousal, while additional activity-related sounds arise from chewing, rooting, movement, mating, play, or regrouping [24,38,40,41,42,43]. These animal-generated sounds coexist with environmental noises from ventilation, feeding equipment, and human or mechanical activity [44,45,46,47,48,49,50], often overlapping in frequency ranges with pig vocalizations [21,40,51]. Consequently, although sound-based monitoring shows strong potential, challenges remain in separating overlapping sound sources, managing environmental variability, and ensuring robust performance across diverse farm conditions [10,27,28,29,30,52].

## 4. Fundamental Concepts of Audio Classification

Audio classification refers to the automatic process of assigning audio signals—such as speech, music, or environmental sounds—to predefined categories using computational methods. It integrates principles from signal processing, feature engineering, and machine learning to enable a wide range of applications, including speech recognition, bioacoustic monitoring, and environmental sound analysis. At its core, audio classification seeks to transform complex, continuous audio waveforms into structured information that can be reliably interpreted by algorithms, even in the presence of noise and variability commonly found in real-world recording conditions [53].

A fundamental step in audio classification is the representation of audio signals in forms that are informative and computationally manageable. While raw waveforms can be directly used as one-dimensional arrays, they are rarely employed due to their high dimensionality and sensitivity to noise [53]. Instead, time–frequency representations such as spectrograms, including Short-Time Fourier Transform (STFT) and Mel-spectrograms, are widely adopted because they describe how frequency components evolve over time and are well suited for both human interpretation and machine learning models, particularly convolutional neural networks [54,55]. Other compact and perceptually motivated representations, such as Mel-Frequency Cepstral Coefficients (MFCCs), Linear Predictive Coding (LPC), and wavelet transforms, are also commonly used to capture salient acoustic characteristics relevant to classification tasks [56,57,58].

Feature extraction further refines these representations by quantifying key properties of the audio signal. Time-domain features, such as zero-crossing rate, short-term energy, and waveform statistics, describe signal amplitude variations over time [59,60]. Frequency-domain features, including spectral centroid, spectral flux, spectral entropy, and MFCCs, characterize the distribution and dynamics of spectral energy and are particularly effective for speech and music analysis [56,59]. Time–frequency features derived from STFT, wavelet transforms, or spectrogram-based methods combine temporal and spectral information, enabling more robust modeling of non-stationary audio signals [53,58,60]. In recent approaches, advanced or hybrid feature strategies are increasingly used, either by combining multiple handcrafted features or by allowing deep learning models to automatically learn discriminative features directly from data, often using spectrogram inputs [54,55,57,61].

Once features are extracted, classification algorithms are applied to assign audio samples to their respective classes. Traditional machine learning methods such as Support Vector Machines, Random Forests, k-Nearest Neighbors, Gaussian Mixture Models, and Decision Trees remain effective for smaller datasets or scenarios with carefully engineered features [56,60]. However, deep learning models—including Convolutional Neural Networks, Recurrent Neural Networks, Transformers, and hybrid CNN–RNN architectures—have become the state of the art for large-scale and complex audio classification tasks due to their ability to learn hierarchical and temporal patterns directly from data [54,55,57,61]. Model performance is typically assessed using evaluation metrics such as accuracy, precision, recall, F1-score, and the area under the ROC curve, following a standard workflow that includes preprocessing, feature extraction, model training with labeled data, and evaluation on unseen datasets [53,54,62].

## 5. Audio Classification Challenges Specific to Pig Farms

Automatic audio classification in pig farms faces several significant challenges that can interrupt or reduce the accuracy and reliability of these systems. The main obstacles include environmental noise, overlapping sounds, data limitations, and variability in farm conditions. Background noise from machinery, ventilation, feeding systems, and human activity can mask or distort pig vocalizations, making it difficult for algorithms to isolate relevant animal sounds [10,40,47]. Overlapping vocalizations from multiple pigs, especially in group housing, create complex audio mixtures that are hard to separate and classify accurately [27,63]. Reverberation and acoustic variability within barns further complicate sound detection and classification [40]. Lightweight deep learning models such as MobileNet and multi-stage ensemble frameworks (e.g., PVMC) have demonstrated high robustness in noisy farm environments, achieving accuracies above 98% and signal-to-noise ratio improvements of up to 4.9 dB [64,65]. Feature-based approaches exploiting formant structure, frequency content, power, and duration further help distinguish pig vocalizations from background sounds [38]. In addition, multi-feature fusion with deep CNNs and attention-based models such as Audio Spectrogram Transformers (AST) improves noise separation and classification performance under real farm conditions [49,52].

Manual labeling of pig sounds is labor-intensive and time-consuming, leading to limited labeled datasets for training robust models [10,49,66]. Lack of standardized, open-access datasets hinders model development and benchmarking across studies and farms [41]. Difficulty in capturing diverse sounds across different pig ages, breeds, and farm environments limits generalizability [66]. Many studies focus on improving algorithms, but less attention is given to optimizing feature extraction and sound labeling, which are critical for performance [40]. Real-time processing on low-cost or embedded hardware can limit model complexity and accuracy [47,67]. Models trained in controlled or specific environments may not perform well in commercial, variable farm settings [27,47].

Privacy issues associated with audio monitoring in pig farms primarily involve data ownership, unauthorized access, and the sharing of sensitive farm-related information with third parties. Although audio-based systems are designed to capture animal vocalizations, recordings may inadvertently include environmental sounds or human conversations, raising potential privacy concerns for farmers. In addition, ambiguities in legal frameworks and data-sharing agreements can create uncertainty regarding how audio data are processed, stored, and used, which may hinder technology adoption. Ensuring farmer privacy requires clear data governance policies, robust data protection practices, and transparent collaboration among farmers, technology providers, and regulators. While the reviewed studies emphasize the benefits of audio monitoring for animal welfare and farm management, explicit consideration of privacy and data protection remains limited, highlighting an important area for future research and guideline development [10,11,68].

## 6. Sound Acquisition Technologies in Pig Farms

Pig farms utilize a range of microphones, from simple unidirectional and omnidirectional types to more advanced microphone arrays and autonomous recording units, depending on specific monitoring objectives. Microphones with a high signal-to-noise ratio (SNR) are particularly important in noisy farm environments, as they improve the reliability and accuracy of sound detection and classification [69]. In practice, the choice of sound acquisition technology depends on the monitoring objective, farm layout, and environmental noise level. Omnidirectional and MEMS microphones are commonly used for general barn-level monitoring due to their low cost and ease of deployment, whereas unidirectional microphones and arrays are preferred when higher spatial resolution or noise suppression is required [10,69,70]. Autonomous recording units enable long-term, unattended data collection and are increasingly adopted in precision livestock farming systems, while wearable microphones remain largely confined to experimental settings due to animal welfare and practicality constraints [70,71,72,73,74]. Commonly used microphones with their applications are listed in Table 1.

In commercial pig barns, microphones are exposed to harsh environmental conditions, including high dust loads, humidity, ammonia, and temperature fluctuations, which can shorten sensor lifespan and degrade recording quality. Sound-based technologies in pig farming emphasize non-invasive, continuously operating microphones that can endure farm environments for long-term monitoring [10,75]. Robustness often involves protective casings or coatings that shield microphones from dust, humidity, and mechanical damage, although exact materials or models are not specified in the literature reviewed. Lightweight wireless microphone systems have been successfully used in animal studies, suggesting that compact, well-protected designs can minimize disturbance and maintain functionality over extended periods [76]. Consequently, durable microphone types such as MEMS microphones and weatherproof autonomous recording units (ARUs) are preferred for long-term deployment, as they are more resistant to dust, moisture, and mechanical stress compared with conventional studio-grade microphones [69,71]. Ceiling-mounted or enclosed installations further reduce direct exposure to animals and contaminants, improving operational longevity in commercial settings [10,73].

The available research identifies very few commercial audio classification products specifically developed for pig farms. Most studies focus on custom or experimental systems, but one notable commercial product is described below. A list of commercially available audio classification systems developed for pig farms is provided in Table 2.

## 7. Audio Annotation and Labeling Platforms

In audio-based monitoring of pig farms, preprocessing is a critical step to ensure reliable feature extraction and robust model performance under noisy and variable recording conditions. Environmental noise originating from machinery, ventilation systems, human activity, and other animals is commonly addressed using signal processing techniques such as discrete wavelet transform, ensemble empirical mode decomposition, independent component analysis, and wavelet threshold denoising, which aim to suppress noise while preserving salient vocal characteristics [24,80,81]. More recently, deep-learning-based denoising frameworks, including animal-independent toolkits such as ANIMAL-CLEAN, have been introduced to further enhance signal quality [82]. Following noise reduction, audio recordings are segmented to retain only informative regions, often through voice activity detection to identify pig vocalizations and exclude silence or irrelevant background sounds, while manual or semi-automatic tools are used to slice recordings into standardized clip lengths (e.g., 2–3 s) suitable for model input [40,41,47]. Amplitude normalization, commonly applied using dBFS, and standardization of sample duration help ensure consistency across datasets and improve model robustness [40,41,49]. High-quality annotation remains essential for supervised learning, with manual labeling by trained personnel or experts frequently employed to classify vocalizations by type or context, sometimes supported by synchronized audio–video cross-checking [40,41]. To address data scarcity and enhance generalization, data augmentation techniques such as pitch shifting, time stretching, temporal shifting, and background noise addition are widely applied to simulate real-world variability encountered in commercial pig farm environments [83,84].

A variety of audio labeling platforms are used in farm animal research and precision livestock farming to support the annotation of vocalizations for supervised and semi-supervised machine learning. Most of these platforms are open-source and highly modifiable, allowing researchers to adapt workflows to different species, recording conditions, and research objectives [85,86,87]. Recent developments increasingly incorporate machine learning–assisted annotation, which improves efficiency and scalability when handling large audio datasets typical of commercial farm environments [87,88]. While many tools are designed for general bioacoustic applications, some platforms and custom solutions have been developed for specific farm animals, such as cattle and goats, offering tailored interfaces and annotation schemes [89,90]. In addition, collaborative and web-based platforms enable multi-user annotation, data sharing, and large-scale project management, which are essential for building high-quality labeled datasets in precision livestock farming [88,91]. Commonly used audio labeling platforms are presented in Appendix A, Table A1.

## 8. Embedded Edge Computing Devices for On-Farm Audio Analysis

Modern farms increasingly rely on embedded and edge computing devices for real-time sound acquisition and analysis, enabling early detection of health, welfare, and behavioral anomalies directly at the farm level [92,93,94,95,96]. Commonly used hardware platforms include embedded AI boards, microcontrollers, and integrated sensor systems designed for low-cost, robust, and scalable deployment in commercial environments. Embedded AI platforms such as NVIDIA Jetson TX2 and Jetson Nano support on-device preprocessing and deep-learning-based anomaly or vocalization detection, offering a balance between computational performance and affordability for small to medium-scale farms [47,97]. Low-power microcontroller platforms, including ESP32-WROOM, are widely adopted in open-source and wireless monitoring frameworks, particularly in rural or resource-limited settings [98]. Recent advances in TinyML have further enabled convolutional neural networks for pig vocalization classification on highly resource-constrained hardware, achieving competitive accuracy with minimal energy and memory requirements [67]. In parallel, custom sensor boards integrated into wearable systems, such as ear tags or collars, facilitate individual-level monitoring, while commercial solutions including SoundTalks^®^ and PILLAR CM-5010Pro provide turnkey systems for continuous respiratory and behavioral surveillance in commercial pig farms [6,10,11,31]. Key edge devices and embedded hardware used for real-time sound acquisition and analysis are presented in Table 3.

## 9. Audio Feature Engineering for Pig Sound Recognition

Audio feature engineering plays a central role in pig sound recognition and has evolved substantially with advances in precision livestock farming. Early studies primarily relied on handcrafted acoustic features such as Mel-Frequency Cepstral Coefficients, short-time energy, zero-crossing rate, and spectral descriptors, which remain computationally efficient and effective for basic classification of coughs, grunts, and screams [24,26,38,100,101]. These features have been complemented by bio-acoustic and domain-specific representations, including time–frequency representations such as Short-Time Fourier Transform, Constant-Q Transform, and spectrograms, as well as formant structure, variability, and sub-band spectrum centroid features, which enhance sensitivity to stress- and disease-related vocal changes [102,103,104]. While these handcrafted and bio-acoustic features are well understood and widely validated, their performance can degrade in noisy, real-world farm environments, motivating the adoption of more adaptive and data-driven feature extraction approaches [100].

Recent research increasingly emphasizes deep learned and hybrid feature representations, which have significantly improved recognition accuracy and robustness. Convolutional neural networks and transformer-based models are now widely used to automatically extract high-level discriminative features from spectrograms or raw audio, often outperforming traditional methods in tasks such as pig cough and abnormal sound detection [30,41,52,105]. Hybrid architectures, including CNN-based networks with attention mechanisms, DenseNet variants, DNN–HMM models, and lightweight MobileNet or TinyML implementations, further enable continuous and real-time monitoring on embedded and edge devices [47,67,106,107,108]. Moreover, feature fusion strategies—such as early and late fusion of handcrafted, deep, and visual features, as well as heterogeneous integration of acoustic data with physiological information like thermal imaging—have consistently yielded superior performance, with reported accuracies exceeding 97% in several studies [28,29,103,109,110,111]. Together, these developments highlight a clear progression from handcrafted to deep learned and fused feature engineering approaches, underpinning the growing effectiveness of automatic pig sound recognition systems for health and welfare monitoring. Summary of key claims and supporting evidence in pig sound feature engineering is presented in Table 4.

## 10. Machine Learning and Deep Learning Approaches for Pig Audio Classification

Machine learning and deep learning approaches have become central to pig audio classification, enabling automatic monitoring of health, welfare, and behavior through the analysis of vocalizations such as coughs, grunts, and screams. Early studies relied on traditional supervised learning algorithms, including support vector machines, decision trees, k-nearest neighbors, and hidden Markov models, typically combined with handcrafted acoustic features such as MFCCs, formants, and spectral descriptors [31,112,113,114,115]. These approaches provided a solid methodological foundation and achieved moderate to high accuracy under controlled conditions; however, their performance was often limited by sensitivity to background noise, environmental variability, and their dependence on manually engineered features [30,101]. Subsequent improvements, such as hybrid DNN–HMM frameworks, demonstrated enhanced sequential modeling and modest gains in accuracy, but still fell short in complex, real-world farm environments [41,101].

In recent years, deep learning has driven substantial performance gains in pig audio classification. Convolutional neural networks are widely used to learn discriminative features directly from spectrograms or raw audio, achieving accuracies approaching or exceeding 99% in tasks such as cough detection and vocalization classification [116,117]. Transformer-based architectures and hybrid CNN–transformer models further improve robustness by capturing both local and global temporal dependencies, with reported accuracies of up to 96% in challenging farm conditions [40,41,52]. Lightweight and hybrid deep models, including MobileNet, ResNet variants, and DNN–HMM systems, enable real-time inference and deployment on embedded and edge devices, while TinyML-based implementations have demonstrated over 90% accuracy on resource-constrained hardware suitable for on-farm monitoring [67,81,106,117]. These deep learning approaches consistently outperform traditional machine learning methods in terms of accuracy, scalability, and resilience to noise [24,26,28,29,64,106].

Beyond model architecture, performance improvements are strongly linked to feature engineering and data handling strategies. While handcrafted acoustic features remain useful, particularly for interpretability and low-complexity models, feature fusion approaches that combine handcrafted, deep learned, and spectrogram-based features have proven especially effective, with some studies reporting accuracies above 99% [24,29,49,58]. Data augmentation techniques, including pitch shifting, time stretching, and background noise injection, further enhance model generalization and mitigate data scarcity issues commonly encountered in farm-based recordings [92,116]. Despite these advances, challenges remain, including the need for large, well-labeled datasets, standardized evaluation protocols, and further optimization of models for embedded and edge AI deployment. Future research directions increasingly emphasize multimodal integration, standardized benchmarks, and explainable AI to improve trust and adoption of audio-based monitoring systems in precision livestock farming [40,110,118]. Summary of recent research for audio classification in pigs is presented in Table 5 and Table 6.

## 11. Disease-Focused Audio Classification Research

Recent disease-focused audio classification research demonstrates that machine learning, particularly deep learning and multimodal approaches, enables accurate and practical detection of respiratory diseases in pigs under real farm conditions. One study employed Mel-Frequency Cepstral Coefficients as acoustic features within a two-level machine learning framework, using support vector data description for anomaly detection followed by sparse- representation-based classification to distinguish Postweaning Multisystemic Wasting Syndrome, Porcine Reproductive and Respiratory Syndrome, and Mycoplasma hyopneumoniae, achieving 94% detection accuracy and 91% classification accuracy, even when using low-cost microphones in commercial barns [31]. In large-scale on-farm applications, a continuous AI-driven sound monitoring system (SoundTalks^®^) was shown to reliably track coughing activity and correlate acoustic indicators with qPCR diagnostics for swine influenza A virus, PRRSV, and Actinobacillus pleuropneumoniae, providing early warning of respiratory health deterioration through clear alignment between pathogen load and vocal distress [79]. More recently, multimodal systems combining deep-learning-based cough detection models, including AlexNet, DenseNet, and MnasNet, with video-based pig localization have achieved up to 95% detection accuracy in noisy farm environments, enabling identification of coughing individuals and supporting more precise, real-time disease surveillance and management [124].

## 12. Behavior Recognition Through Acoustics

Recent studies on audio-based pig behavior recognition demonstrate that deep learning and feature-fusion approaches can accurately and efficiently monitor welfare-related behaviors in real farm environments. Lightweight convolutional neural networks deployed on low-cost embedded (TinyML) devices have successfully classified agonistic and social vocalizations with accuracies exceeding 90%, highlighting the feasibility of real-time, on-device behavioral monitoring without reliance on high-performance hardware [67]. To improve robustness and generalization, a deep convolutional neural network using a Mixed-MMCT feature extraction strategy—combining MFCCs, Mel-spectrograms, Chroma, and Tonnetz features—achieved up to 99.67% accuracy in distinguishing pig vocalizations from non-vocalizations across multiple farms, providing a strong foundation for subsequent fine-grained behavior classification such as screaming or grunting [49]. Complementary work combining time- and frequency-domain features, including short-time energy, frequency centroid, formant frequency, and MFCCs, with neural networks optimized via genetic algorithms reported an average classification accuracy of 93.2% for grunting, squealing, and coughing, enabling reliable automatic feedback on pig behavioral states [24]. Beyond discrete behaviors, large-scale analyses of pig vocalizations using neural networks have also achieved 91.5% accuracy in emotional valence classification and 81.5% accuracy in contextual interpretation, underscoring the potential of audio-based systems for continuous, non-invasive welfare assessment based on affective vocal cues [21]. Recent audio-based pig behavior recognition studies are summarized in the Appendix A, Table A2.

## 13. Real-Time, Edge & On-Farm Deployments

Deploying audio classification systems for pigs in real-time, edge, and on-farm environments requires balancing technical performance with practical farm constraints. Successful implementation depends on robust sensing hardware, efficient and noise-resilient models, reliable data acquisition, and user trust. Microphone selection and placement are critical, as high-quality, wide-frequency microphones help reduce distortion and background noise but must be carefully balanced against cost, durability, and power consumption in commercial barns [10,125,126]. Edge computing platforms such as ARM Cortex-M microcontrollers, Raspberry Pi, and Jetson Nano are increasingly used for on-farm deployment, but they require low-latency and energy-efficient inference pipelines, often relying on TinyML approaches or quantized models to meet strict memory and power constraints [92,127].

Model optimization is therefore central to edge deployment, with techniques such as pruning, quantization, and hardware-aware neural architecture search reducing computational load while maintaining accuracy [128,129]. Feature-based representations, including Mel spectrograms and MFCCs, are often preferred over raw audio on edge devices due to their favorable trade-off between accuracy and computational efficiency [126,127]. In parallel, environmental challenges—such as noise from ventilation systems, feeding equipment, and animal movement—necessitate robust preprocessing, noise reduction strategies, and resilient model architectures to ensure reliable performance under real farm conditions [130]. Beyond technical considerations, usability and trust strongly influence adoption: explainable AI outputs, such as interpretable call-type labels and intuitive dashboards, help build farmer confidence and support regulatory compliance, while low false-alarm rates and seamless integration with existing farm management systems are essential for long-term on-farm acceptance [6,92,131]. Recent research summary on real-time audio classification in pig farms is summarized in Table 7, while the key deployment factors for on-farm audio classification is presented in the Appendix A, Table A3.

## 14. Evaluation Strategies

Evaluation of audio classification systems in pig farming relies on rigorous experimental design, careful feature engineering, and robust statistical validation to ensure both accuracy and real-world applicability. High-quality data collection and labeling form the foundation of evaluation, with most studies relying on manual annotation by trained human experts to create ground truth datasets for vocalizations such as coughs and grunts [24,49,77]. To strengthen label reliability, some experiments employ gold-standard validation methods, including induced behaviors (e.g., controlled cough induction) and parallel on-site human observation [77]. Increasingly, datasets are collected from multiple farms and housing conditions to assess model generalizability across environments, although such diversity remains limited in many studies [49].

Evaluation protocols typically combine standardized preprocessing, robust validation strategies, and comprehensive performance metrics. Commonly extracted features include MFCCs, spectrograms, short-time energy, frequency centroid, and formant frequencies, with preprocessing steps such as filtering, normalization, and segmentation into short windows (typically 1–3 s) to enhance signal quality and reduce noise [24,40]. Model robustness is most often assessed using k-fold cross-validation (commonly 5- or 10-fold), while external validation on independent farms or populations—though less frequent—provides stronger evidence of real-world applicability [21,49,77]. Performance is evaluated using metrics such as accuracy, precision, recall, specificity, F1-score, and AUC, with some studies additionally reporting negative predictive value, Matthews correlation coefficient, runtime efficiency, and interpretability analyses to identify influential features [24,40,49,78,132]. Finally, field trials in commercial barns offer critical real-world testing, allowing system outputs to be compared directly with human observation under practical farm conditions [75,77]. Commonly used evaluation strategies for audio classification is presented in Appendix A, Table A4.

## 15. Generalization Challenges

Generalizing audio classification models across different pig houses remains a major challenge due to substantial environmental variability, dataset limitations, and complex acoustic conditions. Pig housing environments contain diverse and unpredictable background noise sources—such as ventilation systems, machinery, human activity, and overlapping animal vocalizations—which vary considerably between farms and over time, making reliable discrimination of target sounds difficult [10]. Differences in recording conditions, including microphone type, placement, sensitivity, and barn layout, further introduce domain shift and reduce model transferability across locations [40]. These challenges are exacerbated by limited and imbalanced datasets, as collecting and annotating large, diverse audio corpora is labor-intensive; consequently, many models are trained on small or homogeneous datasets that do not fully represent real-world variability, leading to overfitting and poor generalization [49]. Manual annotation itself can introduce labeling errors, particularly in noisy or overlapping sound scenarios, which may bias training and evaluation outcomes [40]. In addition, most existing models are trained on data from a single or few farms and thus become overly specialized to specific acoustic profiles, while the lack of open, standardized datasets and feature extraction protocols further hampers cross-study comparison and generalization [49,133]. Finally, real pig houses often involve simultaneous vocalizations from multiple animals, whereas many studies rely on single-animal recordings, making mixed-sound separation and robust classification substantially more complex under practical farm conditions [40,63].

## 16. Gaps and Limitations Identified in Current Research

Despite recent advances, several critical gaps continue to hinder the success and generalizability of pig farm audio classification systems. A major limitation lies in dataset quality and availability, as most existing datasets are relatively small, collected from a limited number of farms or even single pens, and often focus on a narrow range of vocalization types or conditions, which restricts model generalization to new environments and pig populations [49]. Manual annotation further represents a significant bottleneck, as accurate labeling is labor-intensive, time-consuming, and prone to human error or inconsistency, particularly when dealing with overlapping or ambiguous sounds in noisy barn environments [40,134]. These challenges are compounded by the lack of publicly available, standardized datasets and benchmarking protocols, making cross-study comparison and objective performance evaluation difficult [41]. Real-world farm conditions also introduce substantial acoustic complexity, including background noise and simultaneous vocalizations from multiple pigs, which are frequently underrepresented in curated datasets and inadequately addressed by current models [24,40]. From a methodological perspective, models trained under controlled or farm-specific conditions often exhibit poor robustness and adaptability when deployed in new settings, highlighting persistent generalization issues [133]. Reliance on single or limited feature sets can further reduce classification accuracy in variable environments, while insufficient data diversity increases the risk of overfitting, causing models to perform well during training but fail in practical deployment [40,41]. Finally, practical and technical barriers—such as restricted farm access, equipment limitations, difficulties in continuous high-quality data collection, and computational constraints for deploying complex models on edge devices—remain significant obstacles to large-scale, real-time adoption of audio-based monitoring systems in pig farms [49]. Table A5 summarizes recurrent gaps and limitations identified across the reviewed studies. While not all studies explicitly report each limitation, these issues consistently emerged through comparative analysis of datasets, methodologies, and validation strategies.

## 17. Emerging Opportunities for Future AI Models

Recent advances in AI and deep learning have opened several promising opportunities to enhance pig sound classification across data, model architecture, and real-world deployment. At the model level, advanced feature extraction and fusion strategies—such as integrating MFCCs, Mel-spectrograms, Chroma, and Tonnetz features through novel approaches like Mixed-MMCT—have demonstrated significant improvements in classification accuracy and robustness across heterogeneous farm environments [24,49]. Transformer-based and hybrid CNN–Transformer architectures (e.g., TransformerCNN) further strengthen both local and global feature representation, leading to improved generalization and performance on diverse datasets [41]. Model resilience can be further enhanced by incorporating noise-robust training strategies, including data augmentation techniques such as pitch shifting, time stretching, and background noise injection, alongside advanced noise filtering methods tailored to farm acoustics [135]. Progress in this field is also strongly tied to dataset expansion and standardization; the development of large, publicly available, and well-annotated pig sound datasets would enable meaningful benchmarking, facilitate cross-study comparison, and accelerate methodological advances [41]. To address annotation bottlenecks, automatic and semi-automatic labeling frameworks driven by AI offer scalable solutions for dataset curation while reducing human labor and inconsistency [49]. Finally, emerging multimodal and real-time systems—integrating audio with visual or environmental sensor data such as video and temperature—provide richer contextual understanding of pig behavior and health, while advances in edge computing and TinyML enable lightweight, energy-efficient models for continuous, autonomous, on-farm monitoring in practical production settings [41,92].

## 18. Conclusions

This review demonstrates that AI-based audio classification has evolved into a powerful and practical tool for monitoring respiratory health, behavior, and welfare in pig farming. Early machine learning approaches relying on handcrafted acoustic features laid the foundation for sound-based disease detection, but their sensitivity to noise and limited generalizability restricted large-scale adoption. Recent advances in deep learning, particularly convolutional neural networks, transformer-based architectures, and hybrid models have significantly improved detection accuracy, robustness, and scalability, enabling reliable cough detection and respiratory disease monitoring under real farm conditions. Feature fusion strategies, data augmentation, and multimodal integration further enhance performance and resilience to environmental variability.

Despite these advances, several challenges remain. Limited availability of large, standardized, and diverse datasets continues to hinder model generalization across farms. Manual annotation remains labor-intensive and error-prone, while overlapping vocalizations and complex background noise complicate sound classification in commercial barns. Moreover, deploying computationally intensive models on edge devices requires careful optimization to balance accuracy, latency, and energy consumption.

Looking forward, future research should prioritize the development of open, standardized datasets, automatic or semi-automatic labeling pipelines, and explainable AI frameworks to improve transparency and user trust. Multimodal systems that integrate audio with visual or environmental data, along with continued progress in edge and TinyML technologies, offer promising pathways toward fully autonomous, real-time respiratory health monitoring in swine. Collectively, these advances have the potential to transform respiratory disease management in pig production, supporting improved animal welfare, reduced antibiotic use, and more sustainable livestock systems.

## Figures and Tables

**Table 1:** Overview of microphone types and representative models used in audio-based monitoring of pigs for respiratory disease detection and behavioral analysis.

Microphone Type	Description & Use Case	Models/Systems	Ref.
Unidirectional (Cardioid)	Captures sound mainly from one direction, reducing background noise. Used for targeted monitoring (e.g., above pens).	Audio-Technica M260C (Audio-Technica Corp., Tokyo, Japan); Morbo M66 (Morbo Microphones, Bologna, Italy) *	[10]
Omnidirectional (Electret Condenser)	Captures sound from all directions, suitable for general ambient monitoring.	Panasonic WM-61A (Panasonic Corp., Osaka, Japan); PUI Audio electret microphones (PUI Audio Inc., Dayton, OH, USA)	[10,69]
MEMS Microphones	Micro-Electro-Mechanical Systems; small, robust, and suitable for integration in sensor networks.	InvenSense ICS-40720 (InvenSense Inc., San Jose, CA, USA); SPU0410LR5H-QB (Knowles Electronics, Itasca, IL, USA)	[69]
Microphone Arrays	Multiple microphones arranged spatially for sound localization and source separation.	Sorama L642V sound camera (Sorama B.V., Eindhoven, Netherlands); custom research-built arrays (various academic institutions)	[10,70,71,72]
Autonomous Recording Units (ARUs)	Standalone, weatherproof devices for long-term, remote monitoring.	Song Meter SM2 (Wildlife Acoustics Inc., Maynard, MA, USA); MAARU ARU (research prototype, academic development)	[71,72,73]
Wearable/Mountable Microphones	Attached to animals for individual vocalization tracking (mainly in research).	Custom miniature microphones (research prototypes; various locations)	[74]
Digital Recorders/Camcorders	Used for synchronized audio-video monitoring in some studies.	Sony ICD-UX560F (Sony Corp., Tokyo, Japan); JVC GR-DVL520A (JVCKENWOOD Corp., Yokohama, Japan)	[10]

MEMS: Micro-Electro-Mechanical Systems; ARUs: Autonomous Recording Units. * Location provided at country level where manufacturer city information is not consistently reported in the cited literature.

**Table 2:** Commercially available audio classification systems developed for pig farms.

Product Name/Developer	Product Type	Specifications	Limitations	Ref.
PecSmart^®^ (Pecuária Smart S/A, Florianópolis, Santa Catarina, Brazil)	Integrated hardware + analytics service	- Real-time pig cough detection - 16-microphone array - Deep-learning-based (CNN–RNN hybrid) - Extracts MFCC and spectral features - Reported high accuracy (up to 99.6%) - Designed for commercial swine barns	- Requires multi-microphone installation and stable power/network infrastructure - Limited publicly available details on model generalization across farms	[77]
SoundTalks^®^ (SoundTalks NV, Leuven, Flemish Region, Belgium)	Hardware platform with cloud-based service	- Continuous cough monitoring - Ceiling-mounted microphone units - AI-based cough index calculation - Early warning of respiratory disease outbreaks - Widely deployed in commercial pig farms	- Primarily optimized for cough detection rather than broader behavioral classification - Proprietary algorithms limit transparency and explainability	[78,79]
Pig Cough Monitor (Fancom B.V., Panningen, Limburg, Netherlands)	Hardware-based monitoring system	- Automatic cough detection system - Microphone-based continuous monitoring - Provides alarm thresholds for abnormal coughing - Integrated into precision livestock management platforms	- Threshold-based alerts may require farm-specific calibration - Focused on cough events, with limited capability for multi-class sound recognition	[10]

AI: Artificial Intelligence; CNN: Convolutional Neural Network; RNN: Recurrent Neural Network; MFCC: Mel-Frequency Cepstral Coefficients.

**Table 3:** Representative edge and embedded hardware platforms used for real-time audio-based monitoring and analysis in pig farm environments.

Device/Platform	Description & Use Case	Models/Systems	Ref.
Embedded AI Boards	Run real-time sound processing and machine learning models locally.	NVIDIA Jetson TX2, Jetson Nano (NVIDIA Corporation, Santa Clara, CA, USA)	[47,97]
Microcontroller Platforms	Low-power, cost-effective boards for basic sound acquisition and preprocessing.	ESP32-WROOM (Shanghai, China), Arduino (Zurich, Canton of Zurich, Switzerland), Raspberry Pi (Cambridge, England, UK)	[67,96,98]
TinyML Devices	Specialized for running lightweight ML models on resource-constrained hardware.	Edge Impulse-enabled MCUs (San Jose, CA, USA)	[67]
Custom Sensor Boards	Integrated boards with microphones, accelerometers, and wireless modules for wearables or environmental monitoring.	Ear tag sensor boards, multiparameter sensor boards (Various academic/research institutions)	[6,98]
Commercial Audio Sensors	Standalone or networked microphones with onboard processing for continuous monitoring.	PILLAR CM-5010Pro (Suzhou, Jiangsu Province, China), SoundTalks^®^ (Leuven, Flemish Region, Belgium)	[10,11,31]
AIoT Sensor Nodes	Combine acoustic sensing, wireless communication, and edge ML for distributed monitoring.	Custom AIoT platforms	[99]

AI: Artificial Intelligence; ML: Machine Learning; MCU: Microcontroller Unit; TinyML: Tiny Machine Learning (deployment of ML models on microcontrollers and ultra-low-power devices); AIoT: Artificial Intelligence of Things (integration of AI with Internet of Things devices for distributed sensing and decision making).

**Table 4:** Key findings from published studies on feature engineering strategies for pig sound classification.

Claim	Feature Type	Application Task	Model Type	Reasoning	Ref.
Fusion of acoustic and deep features improves cough recognition	Acoustic + Deep (CQT, STFT, CNN)	Pig cough detection	SVM (with feature fusion)	Combining time-frequency and deep features yields robust, high-accuracy classification	[28]
MFCC and Mel spectrograms enhance oestrus detection	MFCC, Mel spectrograms, DWT	Sow oestrus stage classification	MobileViT (lightweight CNN)	MFCC and Mel spectrograms capture key vocal cues, DWT denoises, enabling efficient, accurate detection	[81]
Hybrid deep features and feature selection yield high cough accuracy	MFCC, spectral, proprietary	Pig cough detection	CNN + RNN hybrid	Feature selection and hybrid deep learning models achieve high precision and recall in field conditions	[77]
Spectral + speech features outperform single features	Spectrogram, time-domain, speech	Pig sound state classification	Parallel CNN + RNN + SVM	Dual input leverages both spectral and temporal cues, boosting accuracy for multiple pig states	[40]
Multi-feature fusion (acoustic + visual) outperforms single features	RMS, MFCC, ZCR, LBP, HOG, CQT	Pig cough detection	SVM, RF, KNN	Fusing acoustic and visual features (from spectrograms) increases recognition rates over single domains	[111]
Mixed-MMCT (MFCC, Mel, Chroma, Tonnetz) boosts vocalization detection	MFCC, Mel-spectrogram, Chroma, Tonnetz	Vocalization vs. non-vocalization	Deep CNN	Integrating multiple feature types and data augmentation improves generalization and robustness	[49]
MFCC-CNN fusion outperforms MFCC alone for cough recognition	MFCC + CNN-derived features	Pig cough detection	CNN, SVM	Fusing MFCC with CNN features increases F1-score and accuracy, especially with optimal frame selection	[110]
DNN-HMM with MFCCs excels in continuous cough recognition	MFCC	Continuous cough detection	DNN-HMM	DNN-HMM models with MFCCs reduce word error rate and outperform GMM-HMM in continuous sound environments	[26]
Heterogeneous fusion (acoustic + thermal) achieves highest accuracy	Acoustic + Deep thermal (images)	Pig cough detection	SVM (with feature fusion)	Combining sound and thermal features provides robust, multi-modal representation for cough recognition	[109]
Rule-based features (formant, power, duration) discern pig screams	Formant, power, frequency, duration	Pig scream detection	Rule-based classifier	Physically meaningful features enable explicit, interpretable scream detection in noisy environments	[38]

CQT: Constant-Q Transform; STFT: Short-Time Fourier Transform; CNN: Convolutional Neural Network; RNN: Recurrent Neural Network; SVM: Support Vector Machine; RF: Random Forest; KNN: K-Nearest Neighbors; MFCC: Mel-Frequency Cepstral Coefficients; DWT: Discrete Wavelet Transform; MobileViT: Mobile Vision Transformer (lightweight CNN–Transformer hybrid); RMS: Root Mean Square; ZCR: Zero-Crossing Rate; LBP: Local Binary Patterns; HOG: Histogram of Oriented Gradients; DNN-HMM: Deep Neural Network–Hidden Markov Model; GMM-HMM: Gaussian Mixture Model–Hidden Markov Model.

**Table 5:** Summary of experimental settings and datasets used in representative audio-based pig classification studies.

Sensors Name	Sensor Number	Number of Pigs Used	Age of Pigs Used	Research Duration	Dataset Number	Objectives	Findings	Ref.
Microphone (Sennheiser ME66, Wedemark, Lower Saxony, Germany)	1	411, from 5 previous studies	Birth to slaughter	Not specified (multi-study aggregation)	>38,000 calls (Dataset S1)	Classify pig calls by emotional valence/context	Neural network outperformed pDFA; robust across contexts and ages	[21]
PLM-Q5 noise reduction microphone, Raspberry Pi 4 (Cambridge, England, UK)	1 per pen	~36 (12 per pen, 3 farms)	Not specified	24 h continuous per farm	3 datasets × 4000 files	Classify vocalization vs. non-vocalization	Mixed-MMCT feature extraction improved robustness and accuracy	[49]
Acoustic test analyzer (BK 2270-S-C, 4189 mic, Darmstadt, Hesse, Germany)	1	189	Fattening stage	1 month	Not specified	Classify grunting, squealing, coughing	Multi-feature fusion improved recognition of vocal types	[24]
Directional microphones (Various)	1	24	Piglets	Not specified	Not specified	Classify agonistic/social vocalizations	TinyML feasible for real-time embedded monitoring	[67]
Microphones (SmartMic, PecSmart, Florianópolis, Santa Catarina, Brazil)	1 per pen	256 (16 per pen, 16 pens)	Growing–finishing	6 days	1110 coughs, 8938 other sounds	Detect coughs in field conditions	High performance for cough detection in commercial barns	[77]
Microphone (external iTalk-02)	1	10	Adult Landrace	10 h	Not specified	Classify eating, estrus, howling, humming, panting	DNN-HMM outperformed HMM, GMM-HMM, SVM, ResNet18	[107]
Microphone (TCD-D8, SONY, Tokyo, Japan)	2	70	Young pigs	Not specified	4537 calls	Classify pain-related vocalizations	Screams indicate pain; automatic classification feasible	[119]
Microphone (JVC GR-DVL520A, Yokohama, Kanagawa, Japan)	1	36	25–30 kg	30 min per pig	Not specified	Detect wasting diseases via cough	Acoustic differences in coughs by disease; early detection possible	[120]
Microphone (U.S. Blaster condenser)	1	44	150 days	Not specified	Not specified	Detect coughs in field	Feasible for field cough detection	[121]
Microphone	1	40	Pre-weaning gilts	5 min isolation	14,000+ vocalizations	Identify call types, relate to arousal	Seven call types identified; call type linked to behavior	[122]
Microphone (Yoga^®^, Taipei City, Taiwan)	1	40	22 weeks	Not specified	Not specified	Classify stress (pain, cold, hunger)	Vocalization effective for stress detection	[123]

PLM-Q5: Professional Lavalier Microphone model Q5; BK 2270-S-C: Brüel & Kjær Type 2270 Sound Level Meter (Class C); 4189 mic: Brüel & Kjær Type 4189 Free-field Microphone; pDFA: permuted Discriminant Function Analysis; DNN-HMM: Deep Neural Network–Hidden Markov Model; GMM-HMM: Gaussian Mixture Model–Hidden Markov Model; SVM: Support Vector Machine; TinyML: deployment of machine learning models on ultra-low-power embedded devices; Mixed-MMCT: Mixed Mel-spectrogram, MFCC, Chroma, and Tonnetz feature extraction framework.

**Table 6:** Summary of feature extraction and classification techniques applied in representative audio-based pig classification studies.

Extraction Technique	Classification Technique	Accuracy	Precision	Recall	F1	References
Acoustic features (MFCC, spectral, temporal)	Neural network, pDFA	91.5% (NN, valence), 81.5% (NN, context)	Not specified	Not specified	Not specified	[21]
MFCC, Mel-spectrogram, Chroma, Tonnetz, Mixed-MMCT	Deep CNN (DCNN)	99.5–99.7% (per farm), 95.67% (cross-farm)	96.25%	95.68%	95.96%	[49]
STE, FC, FF, MFCC, PCA	BP neural network (GA optimized)	93.2%	92.9%	92.8%	Not specified	[24]
Not specified	CNN (TinyML, Edge Impulse)	>90%	Not specified	Not specified	Not specified	[67]
34 audio features (MFCC, others)	Hybrid CNN-RNN	99.6%	98.8%	98.6%	98.6%	[77]
MFCC (39-dim), Kalman filter, EMD-TEO	DNN-HMM	83%	Not specified	Not specified	Not specified	[107]
Multiparametric call analysis	Discriminant analysis	94.6% (call type classification)	Not specified	Not specified	Not specified	[119]
MFCC, labeling	SVDD, SRC	94% (detection), 91% (classification)	Not specified	Not specified	Not specified	[120]
Filter bank, amplitude demodulation	Dynamic time warping	85.5% (cough), 86.6% (other)	Not specified	Not specified	Not specified	[121]
Acoustic/spectral features	Manual + statistical classification	Not specified	Not specified	Not specified	Not specified	[122]
Acoustic features	J48 decision tree	81.1%	Not specified	Not specified	Not specified	[123]

MFCC: Mel-Frequency Cepstral Coefficients; STE: Short-Time Energy; FC: Frequency Centroid; FF: Formant Frequency; PCA: Principal Component Analysis; BP Neural Network: Backpropagation Neural Network; GA: Genetic Algorithm; DCNN: Deep Convolutional Neural Network; Mixed-MMCT: Mixed Mel-spectrogram, MFCC, Chroma, and Tonnetz feature extraction framework; pDFA: permuted Discriminant Function Analysis; CNN: Convolutional Neural Network; RNN: Recurrent Neural Network; DNN-HMM: Deep Neural Network–Hidden Markov Model; EMD-TEO: Empirical Mode Decomposition–Teager Energy Operator; SVDD: Support Vector Data Description; SRC: Sparse Representation Classifier; J48: C4.5 decision tree implementation; TinyML: machine learning deployment on ultra-low-power embedded devices. The studies summarized in this table correspond to the experimental datasets described in Table 5; Table 6 focuses specifically on signal processing and classification performance.

**Table 7:** Summary of representative studies on real-time and on-farm audio-based pig classification systems.

Device Used	Objectives	Methodology	Results	Ref.
16 microphones (SmartMic, PecSmart, Florianópolis, Santa Catarina, Brazil)	Detect pig coughs in commercial farm (field validation)	Hybrid deep learning (CNN + RNN); 34 audio features; 10-fold cross-validation; feature selection	High accuracy: 99.6%, recall: 98.6%, F1: 98.6%. Efficient for on-farm cough monitoring.	[77]
Raspberry Pi 4 Model B (Cambridge, England, UK), PLM-Q5 microphone	Classify pig vocalization vs. non-vocalization	Deep CNN; Mixed-MMCT feature extraction (MFCC, Mel-spectrogram, Chroma, Tonnetz); data augmentation	Accuracy: 99.5–99.7% (intra-farm); 95.7% (cross-farm). Robust to new data.	[49]
Audio Spectrogram Transformer (unspecified HW)	Detect abnormal pig vocalizations for welfare monitoring	Audio segmentation; AST model with attention; feature selection; interpretability analysis	Accuracy: 93%; inference speed 19× faster than CNNs; improved efficiency and scalability.	[52]
UM-ASPP-MobileViT (edge-optimized)	Detect sow oestrus via vocalization	MobileViT-based model; DWT denoising; MFCC and Mel spectrogram features; annotated oestrus dataset	Precision: 96.5%, F1: 96.5%; only 1.44 GFLOPs; fast, accurate, and efficient for real-time.	[81]
Lenovo B610 recorder (Beijing, China)	Classify pig vocalizations (sows)	Dual input: spectrogram + time-domain features; parallel network (CNN + custom classifier)	Accuracy: 93.4%, AUC: 0.99; robust in noisy, multi-pig environments.	[40]
NanoPc-T4 (Shenzhen, Guangdong, China), iTalk-02 microphone	Pig sound recognition (behavioral states)	DNN-HMM model; Kalman filtering; MFCC features; empirical mode decomposition for endpoint detection	Accuracy: 83% (custom), 79% (AudioSet); outperforms SVM, ResNet18, GMM-HMM.	[30]
BK 2270-S-C analyzer, 4189 microphone (Darmstadt, Hesse, Germany)	Classify pig grunting, squealing, coughing	Multi-feature fusion (STE, MFCC, formant, etc.); BP neural network; principal component analysis	Avg. accuracy: 93.2%; precision: 87.9–98.1%; recall: 87.4–99.1%.	[24]
Digital camcorder, PC soundcard	Detect pig wasting diseases via cough	MFCC extraction; SVDD for anomaly detection; SRC for disease classification	Detection: 94%, classification: 91%; works with low-cost microphones.	[31]
TransformerCNN (unspecified HW)	Classify domestic pig sounds (behavior/emotion)	Parallel CNN + Transformer; multiple audio features; open-access dataset	Accuracy: 96.1%, AUC: 98.4%, recall: 90.5%; robust and generalizable.	[41]
Audio-visual system (multimodal, unspecified)	Detect coughing pigs and localize individuals	Audio cough detection, video pig detection, multimodal fusion; real farm data; spectrum preprocessing	Detection accuracy: 95%; robust in noisy, real-world farm conditions.	[124]

CNN: Convolutional Neural Network; RNN: Recurrent Neural Network; MFCC: Mel-Frequency Cepstral Coefficients; Mixed-MMCT: Mixed Mel-spectrogram, MFCC, Chroma, and Tonnetz feature extraction framework; AST: Audio Spectrogram Transformer; DWT: Discrete Wavelet Transform; DNN-HMM: Deep Neural Network–Hidden Markov Model; STE: Short-Time Energy; SVDD: Support Vector Data Description; SRC: Sparse Representation Classifier; AUC: Area Under the Receiver Operating Characteristic Curve; GFLOPs: Giga Floating-Point Operations per Second.

## Data Availability

No new data were generated during this study.

## Abbreviations

The following abbreviations are used in this manuscript:

AI	Artificial Intelligence
STFT	Short-Time Fourier Transform
MFCCs	Mel-Frequency Cepstral Coefficients
LPC	Linear Predictive Coding
CNN	Convolutional Neural Network
RNN	Recurrent Neural Network
ROC	Receiver Operating Characteristic
SNR	Signal-to-Noise Ratio
MEMS	Micro-Electro-Mechanical Systems
ML	Machine Learning
MCU	Microcontroller Unit
AIoT	Artificial Intelligence of Things
DNN–HMM	Deep Neural Network–Hidden Markov Model
SVM	Support Vector Machine
CQT	Constant-Q Transform
RF	Random Forest
KNN	K-Nearest Neighbors
DWT	Discrete Wavelet Transform
RMS	Root Mean Square
ZCR	Zero-Crossing Rate
LBP	Local Binary Patterns
HOG	Histogram of Oriented Gradients
GMM-HMM	Gaussian Mixture Model–Hidden Markov Model
STE	Short-Time Energy
FC	Frequency Centroid
FF	Formant Frequency
PCA	Principal Component Analysis
BP Neural Network	Backpropagation Neural Network
GA	Genetic Algorithm
DCNN	Deep Convolutional Neural Network
EMD-TEO	Empirical Mode Decomposition–Teager Energy Operator
SVDD	Support Vector Data Description
PRRSV	Porcine Reproductive and Respiratory Syndrome Virus
AST	Audio Spectrogram Transformer
SRC	Sparse Representation Classifier
AUC	Area Under the Receiver Operating Characteristic Curve

## Appendix A

**Table A1:** Representative audio annotation platforms used for labeling farm animal vocalizations in research and precision livestock applications.

Platform Name	Key Features & Use Cases	Ref.
Crowsetta	Python-based, works with any annotation format, supports flexible workflows for animal vocalizations and bioacoustics data.	[85]
Whombat	Browser-based, user-friendly, supports collaborative annotation, visualization, and ML-assisted workflows.	[91]
ecoSound-web	Open-source, online, supports manual and automatic annotation, peer review, and reference libraries.	[88]
DISCO	Open-source, deep learning ensemble for segmentation and labeling, includes visual tools for analysis.	[87]
NEAL	R/Shiny-based, interactive, designed for large datasets, modifiable for generic or farm animal audio.	[86]
Custom/Semi-automatic Tools	Tailored for specific species (e.g., monophonic cow sound annotation tool, goat vocalization annotation tool), often semi-automatic and visually transparent.	[89,90]

ML: Machine Learning; R/Shiny: R-based interactive web framework; open-source indicates publicly available source code.

**Table A2:** Summary of representative studies on audio-based pig behavior recognition.

Study/Method	Behaviors Detected	Model/Features Used	Accuracy/Findings	Ref.
TinyML CNN	Agonistic, social	CNN, embedded system	>90% accuracy	[67]
DCNN Mixed-MMCT	Vocalization/non-vocalization	DCNN, multi-feature fusion	Up to 99.67% accuracy	[49]
Multi-feature fusion NN	Grunting, squealing, coughing	NN, time/frequency features	93.2% average accuracy	[24]
Neural network on call valence	Emotional valence, context	Neural network, spectrograms	91.5% (valence), 81.5% (context)	[21]

CNN: Convolutional Neural Network; TinyML: deployment of machine learning models on ultra-low-power, resource-constrained embedded devices; DCNN: Deep Convolutional Neural Network; Mixed-MMCT: Mixed Mel-spectrogram, Mel-Frequency Cepstral Coefficients (MFCC), Chroma, and Tonnetz feature extraction framework; NN: Neural Network.

**Table A3:** Critical factors affecting on-farm deployment of audio-based pig monitoring systems.

Factor	Importance/Role	References
Microphone quality	Accurate sound capture, noise reduction	[10,125,126]
Edge device efficiency	Real-time, low-power inference	[92,125,126,127]
Model compression	Fit models to device constraints	[125,127,128,129]
Noise robustness	Reliable detection in farm environments	[10,125,126,130]
Explainable outputs	Farmer trust, regulatory compliance	[92]
Power/connectivity	Continuous, autonomous operation	[10,125]
User adoption	Practicality, integration, low false alarms	[6,131]

**Table A4:** Common evaluation strategies and validation approaches used in audio-based pig sound classification studies.

Strategy	Description/Use Case	References
Manual annotation	Human-labeled ground truth	[24,49,77]
Cross-validation	K-fold (5/10) for robustness	[24,49,77]
External validation	Testing on new farms/populations	[21,49]
Standard metrics	Accuracy, precision, recall, F1, AUC	[24,40,49,78]
Field trials	Real-world deployment and comparison	[75,77]

AUC: Area Under the Receiver Operating Characteristic Curve; K-fold cross-validation: a resampling method where data are split into K subsets (commonly 5 or 10) to assess model robustness and reduce overfitting.

**Table A5:** Synthesized gaps and limitations in current audio-based pig sound research, as identified through comparative analysis of the reviewed literature.

Limitation	Description/Impact	References
Dataset diversity	Small, non-standardized, limited environments	[24,40,41,49]
Annotation challenges	Labor-intensive, error-prone, inconsistent	[40,41,49,134]
Generalization	Poor transfer to new farms/environments	[40,49,133]
Overlapping sounds	Rarely addressed in model design	[40]
Real-world noise	Underrepresented in curated datasets	[24,40,49]
